# Nonsurgical Endodontics and Decompression-Based Management of Extensive Periapical Cystic-Like Lesions: A Comparative and Radiological Study with A Two-Year Follow-Up

**DOI:** 10.3390/jcm14176127

**Published:** 2025-08-29

**Authors:** Roxana Talpoș-Niculescu, Ioana Veja, Carina Sonia Neagu, Laura Cristina Rusu, Șerban Talpoș-Niculescu, Mălina Popa, Luminița Maria Nica

**Affiliations:** 1 Discipline of Restorative Dentistry and Endodontics, Research Center TADERP, Faculty of Dental Medicine, “Victor Babes” University of Medicine and Pharmacy, 300041 Timisoara, Romania; clinci.roxana@umft.ro (R.T.-N.); carina.neagu@umft.ro (C.S.N.); nica.luminita@umft.ro (L.M.N.); 2 Department of Dental Medicine, Faculty of Dentistry, “Vasile Goldiș”, Western University of Arad, Str. Liviu Rebreanu 86, 310045 Arad, Romania; veja.ioana@uvvg.ro; 3 Discipline of Oral Pathology, Multidisciplinary Center for Research, Evaluation, Diagnosis and Therapies in Oral Medicine, Faculty of Dental Medicine, “Victor Babes” University of Medicine and Pharmacy, 300041 Timisoara, Romania; laura.rusu@umft.ro; 4 Discipline of Oral and Maxillo-Facial Surgery, Faculty of Dental Medicine, “Victor Babes” University of Medicine and Pharmacy, 300062 Timisoara, Romania; 5 Discipline of Pedodontics, Pediatric Dentistry Research Center, Faculty of Dental Medicine, “Victor Babes” University of Medicine and Pharmacy, 300041 Timisoara, Romania

**Keywords:** endodontics, periapical lesion, decompression, CBCT, radicular cyst, conservative treatment

## Abstract

**Background:** Large periapical cyst-like lesions pose challenges for nonsurgical management. This study evaluated the clinical and radiological outcomes of conventional endodontic therapy alone versus endodontic therapy with decompression in treating such lesions. **Methods:** Ten patients with CBCT-confirmed periapical lesions >5 mm were randomly assigned to two groups. Group 1 received standard root canal treatment with calcium hydroxide; Group 2 received the same protocol plus decompression. Lesion dimensions were measured in three planes using CBCT at baseline, 12 months, and 24 months. **Results:** Both groups showed significant lesion size reduction over time. At 12 months, Group 2 had a significantly greater mean reduction (8.49 ± 5.09 mm) compared to Group 1 (4.36 ± 1.46 mm) (*p* = 0.008). At 24 months, both groups had comparable outcomes (Group 1: 1.12 ± 0.98 mm; Group 2: 2.41 ± 5.15 mm; *p* = 0.356), with most lesions nearly or fully resolved. Histopathology confirmed radicular cysts in decompressed cases. **Conclusions:** Conservative endodontic therapy is effective for large periapical lesions. Adjunctive decompression accelerates early healing, particularly in extensive cases, potentially reducing the need for surgical intervention. CBCT is valuable for monitoring healing.

## 1. Introduction

Large periapical lesions of endodontic origin, particularly those exhibiting cyst-like radiographic appearances, remain among the most complex clinical entities encountered in contemporary endodontics. Their size, potential for epithelization, and interference with obturation—especially due to persistent intracanal exudate—can compromise the prognosis of nonsurgical root canal therapy. These lesions typically arise from chronic inflammation following pulp necrosis and bacterial invasion of the root canal system, culminating in progressive bone resorption and the formation of radiolucent defects visible on radiographs, including CBCT scans [1]. Histologically, periapical lesions may present as granulomas and cysts, with radicular cysts representing approximately 15–42% of all periapical lesions [2]. Within this category, two subtypes are described, true cysts (closed cysts), which are completely enclosed by an epithelial lining and lack communication with the root canal system, and bay cysts (pocket cysts), which retain a direct connection with the canal space [3]. Although these features may occasionally be suggested radiographically, especially with CBCT imaging, there are no significant clinical, radiographic, histopathologic, or bacteriologic differences between the two types, aside from their morphological relationship with the root canal system. Both are associated with intra-radicular (and occasionally extra-radicular) infection [4,5].

However, this differentiation can only be made reliably through histopathological examination of biopsy specimens, which in the present study was available only in decompression cases; it cannot be determined during standard root canal treatment when no tissue sample is obtained.

The traditional assumption that true cysts are self-sustaining and independent of root canal infection has been questioned. A 2020 study by Ricucci et al. [4] concluded that both cyst types respond favorably to conservative endodontic therapy focused on the elimination of intracanal bacterial infections. As such, the distinction between true and bay cysts may be unnecessary for clinical decision-making or treatment planning [4].

Traditional paradigms in endodontics have long held that lesions larger than 10 mm, particularly those suspected of being cystic in nature, require surgical management, such as apical curettage or enucleation, to achieve resolution [2,6]. Nevertheless, recent clinical reports and systematic reviews suggest that even extensive periapical lesions can respond favorably to conservative treatment modalities—especially when meticulous disinfection protocols and intracanal medicaments such as calcium hydroxide are employed [7,8,9,10]. This has contributed to a shift toward more biologically oriented, minimally invasive strategies aimed at preserving periapical structures and avoiding unnecessary surgery.

One such adjunctive conservative method is decompression, a minimally invasive technique that facilitates intracystic drainage, reduces internal pressure, and promotes regression of the lesion over time. Decompression has gained traction in both endodontics and maxillofacial surgery as a low-risk intervention that can serve as an alternative to immediate surgical excision. Additionally, the decompression procedure allows for the collection of tissue specimens for histopathological confirmation, enabling a definitive diagnosis between granulomatous and cystic lesions—an important step for treatment planning and prognosis estimation [11,12,13,14].

In parallel, the evolution of imaging technology, particularly cone-beam computed tomography (CBCT), has markedly improved the clinician’s ability to diagnose, plan, and monitor periapical pathology. CBCT provides high-resolution three-dimensional imaging that surpasses traditional periapical radiographs in sensitivity, especially for large or irregular lesions. It also permits volumetric and spatial assessment of healing, allowing clinicians to track lesion regression with increased precision [15,16,17,18].

Despite these technological and conceptual advancements, limited prospective data exist comparing the clinical outcomes of conventional endodontic therapy alone versus endodontic therapy combined with decompression for the management of large periapical cyst-like lesions. Much of the existing literature consists of retrospective analyses or isolated case reports with variable follow-up durations and heterogeneous measurement protocols. Furthermore, few studies have incorporated CBCT-based lesion measurement in multiple planes or histological validation of cystic lesions, which are critical for the reliable interpretation of outcomes.

Therefore, the aim of this study was to evaluate and compare the clinical and radiological healing of extensive periapical cyst-like lesions treated with two nonsurgical approaches: (1) standard root canal therapy using calcium hydroxide as an intracanal medicament, and (2) the same protocol supplemented with decompression. Healing was assessed through CBCT measurements at baseline, 12 months, and 24 months, and histopathological confirmation was obtained in decompression cases where tissue was available. By addressing the limitations of previous reports and incorporating standardized diagnostic, therapeutic, and follow-up protocols, this study seeks to contribute robust evidence supporting conservative management strategies for large periapical lesions and to refine clinical decision-making in cases traditionally considered surgical.

## 2. Materials and Methods

### 2.1. Patient Selection

Patients were recruited from the Department of Endodontics at the “Victor Babeș” University of Medicine and Pharmacy in Timișoara, Romania, between May 2020 and December 2022. All eligible individuals presenting with chronic apical periodontitis and CBCT-confirmed periapical lesions larger than 5 mm in at least one dimension were considered for inclusion. The study was conducted in full compliance with the Declaration of Helsinki and was approved by the Institutional Ethics Committee of the “Victor Babeș” University of Medicine and Pharmacy Timișoara (approval number 100/01.05.2020, revised 2025). Written informed consent was obtained from all participants.

The sample size of ten patients (*n* = 10) was chosen based on the exploratory nature of this pilot clinical study, aimed at providing preliminary data for future, larger-scale investigations. This number also allowed for a manageable and standardized follow-up across a two-year period, with high adherence and minimal attrition.

Inclusion criteria were as follows: (1) presence of a periapical lesion ≥5 mm in at least one dimension on CBCT, (2) teeth eligible for primary or secondary nonsurgical endodontic treatment, and (3) absence of systemic conditions contraindicating dental treatment. Exclusion criteria included non-restorable teeth, lesions of non-endodontic origin, or prior surgical treatment of the affected area.

At baseline, periapical lesions were identified and characterized using CBCT. Lesions exhibiting a well-circumscribed radiolucent area exceeding 5 mm in at least one dimension, often with corticated borders and displacement of adjacent anatomical structures, were provisionally described as ‘cyst-like’ based on radiographic appearance. However, as CBCT cannot reliably distinguish a granuloma from a cyst, all such findings are referred to in this study as periapical radiolucencies (PARLs) unless histopathological confirmation was obtained. The designation ‘radicular cyst’ is used exclusively for cases where biopsy analysis confirmed this diagnosis.

### 2.2. Treatment Groups

The study included 10 patients (4 males, 6 females) aged 22–54 years (mean age 36.8 ± 9.4 years). Teeth involved were distributed as follows: maxillary anterior (*n* = 3), maxillary premolars (*n* = 2), mandibular premolars (*n* = 2), and mandibular molars (*n* = 3). All cases presented with non-vital pulps and periapical radiolucencies measuring ≥5 mm in at least one dimension on CBCT. At baseline, the mean maximum lesion dimension was 7.3 ± 1.74 mm in Group 1 and 13.7 ± 3.34 mm in Group 2.

All patients underwent comprehensive clinical assessment, including vitality testing, percussion, palpation, and periodontal probing, followed by CBCT evaluation for radiographic diagnosis and treatment planning. Based on the diagnostic findings, a treatment plan was developed for either primary nonsurgical root canal therapy or nonsurgical retreatment, as appropriate. Ten patients with large periapical lesions (>5 mm on CBCT) were selected from the university dental clinic. All initially received standardized nonsurgical endodontic treatment with calcium hydroxide dressing. Based on the clinical response—specifically the ability to achieve canal dryness—patients were allocated to two groups (*n* = 5 per group): those who responded favorably (Group 1) and those requiring additional decompression due to persistent exudation (Group 2).

Group 1 (conventional treatment): Root canal therapy was performed using calcium hydroxide as intracanal medicament. Due to persistent moisture, obturation was delayed by three weeks.

**Figure 1:**
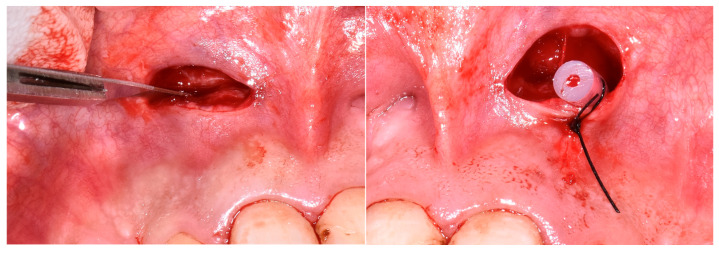
The incision made in the mobile buccal mucosa using a no. 15 Bard-Parker surgical blade and the drainage tube placed into the surgically created window and sutured to the mucosal margins.

Group 2 (decompression group): Underwent the same protocol as Group 1, but with adjunctive decompression. A small incision was made in the buccal mucosa, a drain inserted to allow cystic drainage for 48 h, followed by completion of endodontic therapy two weeks later. At the second appointment (after 3 weeks), calcium hydroxide was removed, and canals were re-irrigated; however, persistent serous exudate was observed. As adequate drying remained unattainable, calcium hydroxide was re-applied, and the tooth was temporarily restored. During the same session, decompression was performed under local anesthesia. A small incision was made in the mobile buccal mucosa using a No. 15 Bard-Parker surgical blade, penetrating the cystic lining. A drainage tube was inserted through the buccal cortical plate and sutured at the mucosal margins to allow for continuous evacuation of the lesion content. The drain was removed after 48 h. Endodontic treatment was completed approximately two weeks later, once secretion had resolved (Figure 1).

### 2.3. Root Canal Treatment Protocol

All procedures were performed by the same experienced endodontist under magnification using an operating microscope (CJ-Optik, Etzersdorf, Germany) and rubber dam isolation. Working length was established using an electronic apex locator (Root ZX II, J. Morita, Kyoto, Japan) and confirmed radiographically. Apical patency was maintained throughout instrumentation using a size 10 K-file.

Mechanical preparation was carried out with the WaveOne^®^ Gold reciprocating file system (Dentsply Sirona, Ballaigues, Switzerland), typically to a final size of Primary (25/07) or Medium (35/06) depending on canal anatomy and apical diameter, achieving a continuous taper to working length.

Abundant irrigation with 5.25% sodium hypochlorite (NaOCl) solution (Chloraxid 5.25%, CERKAMED, Wojciech Pawlowski, Stalowa Wola, Poland), alternated with 17% ethylenediaminetetraacetic acid (EDTA) solution (Endo-Solution 17%, CERKAMED), was performed throughout instrumentation. Final irrigation was activated ultrasonically using IrriSafe™ tip K25 (Acteon Satelec, Mérignac, France) connected to a piezoelectric ultrasound unit (Satelex Suprasson P5 Booster, Acteon Satelec) at low power (3, green; 30 kHz), for 30 s.

Calcium hydroxide paste (Calcipast^®^, CERKAMED, Poland) was placed as an intracanal medicament using a lentulo spiral to working length. At the subsequent visit, medicament was removed by copious irrigation with NaOCl, followed by EDTA, with ultrasonic activation as described above, and final rinse with distilled water.

Final obturation was performed using warm vertical compaction of gutta-percha with the Continuous Wave technique, employing the EQ-V obturation system (Meta Biomed^®^ Europe GmbH, Essen, Germany). WaveOne^®^ Gold Gutta-Percha cones, matching the final preparation size, were compacted in combination with a resin-based root canal sealer (Syntex, CERKAMED, Poland).

In cases where persistent exudate prevented canal drying after calcium hydroxide therapy, decompression was indicated. The procedure was performed by creating a small vestibular window using a sterile scalpel blade no. 15. After creating access to the lesion, the area was gently irrigated with a metronidazole solution (Metronidazole 0.5% B. Braun Melsungen AG, Melsungen, Germany). A soft rubber drain tube was inserted through the surgical window and positioned within the lesion to facilitate decompression. The mucosal margins were sutured around the tube to secure it in place. The drain was maintained for 48 h, after which it was removed. At the following appointment, when the root canal was re-accessed, canal frying could be achieved, and tridimensional obturation was performed successfully using warm vertical compaction.

Coronal sealing was performed directly with composite resin (Asteria, Tokuyama Dental Corp., Tokyo, Japan) in the same session as obturation. In cases requiring adhesive cementation of a fiber post, glass fiber posts (Reforpost, Angelus, Londrina, PR, Brazil) were cemented using Panavia SA Cement (Kuraray Noritake Dental Inc., Tokyo, Japan). When full coverage was indicated, indirect restorations were provided within three months of obturation.

### 2.4. CBCT Measurement Protocol

Radiological assessment was conducted using the OP 3D™ Pro CBCT unit (KaVo Dental GmbH, Biberach, Germany) and measurements were performed using OnDemand3D™ software (version 1.1 (build 1.0.10.1007) Cybermed Inc., Seoul, Republic of Korea). Lesion dimensions were recorded in three planes—mesiodistal, buccolingual, and vertical (height)—using the software’s built-in linear measurement tools. The same operator performed all measurements under standardized conditions, using a calibrated high-resolution monitor. For reliability, a second examiner independently repeated 30% of the measurements. Intraclass correlation coefficients (ICCs) were calculated, yielding values >0.90 for all measurements, indicating excellent inter-observer agreement. The mean of two repeated measurements was used for analysis. The CBCT voxel size was 0.2 mm, and slice thickness was 1 mm.

Clinical, radiological (CBCT), and, where applicable, histological evaluations were conducted at baseline, 1 year, and 2 years post-treatment. Histological analysis was performed when tissue samples were available during decompression.

### 2.5. Statistical Analysis

All statistical analyses were performed using MedCalc Statistical Software version 22.009 (MedCalc Software Ltd., Ostend, Belgium). Normality was confirmed using Kolmogorov–Smirnov, Shapiro–Wilk, and Anderson–Darling tests. Independent *t*-tests were used for intergroup comparisons; paired *t*-tests for intragroup comparisons. Levene’s test and Welch’s correction were applied when needed. Significance threshold was set at α = 0.05. Cohen’s d and 95% CI were reported.

## 3. Results

All periapical lesion dimensions were assessed on CBCT scans in three orthogonal planes (mesiodistal, buccolingual, and vertical) using dedicated software (OnDemand3D™ integrated with the OP 3D™ Pro CBCT system). Measurements were performed by a single calibrated examiner, with a second examiner reviewing 30% of the scans. The measurement procedure proved highly reliable: inter-observer intraclass correlation coefficients (ICCs) exceeded 0.90 and intra-observer ICC values were above 0.95, indicating excellent consistency.

**Figure 2:**
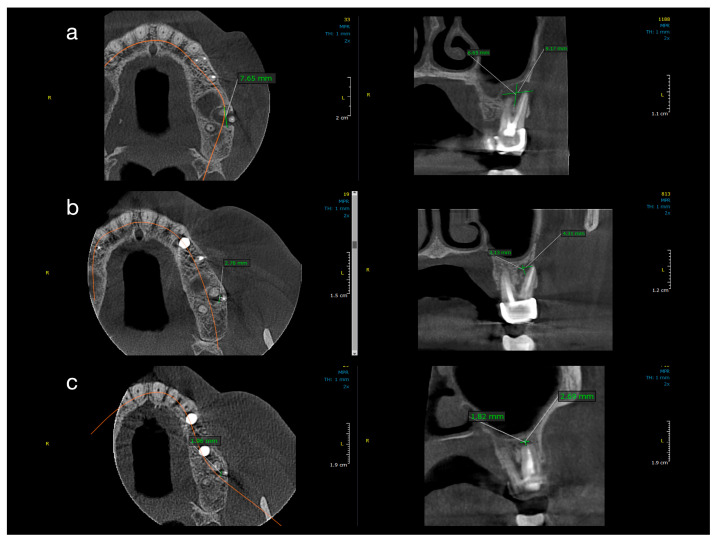
Sequential CBCT images of a case from Group 1 (conventional treatment). Axial, sagittal, and coronal CBCT slices showing lesion reduction in a patient treated with conventional root canal therapy and calcium hydroxide. (**a**) Baseline: well-defined radiolucency measuring 7.65 mm mesiodistally, 6.55 mm buccolingually, and 9.17 mm vertically. (**b**) One-year follow-up: partial healing with reduction to 2.78 mm (MD), 3.13 mm (BL), and 4.3 mm (V). (**c**) Two-year follow-up: near-complete resolution (1.96 mm MD, 1.82 mm BL, 2.69 mm V).

**Figure 3:**
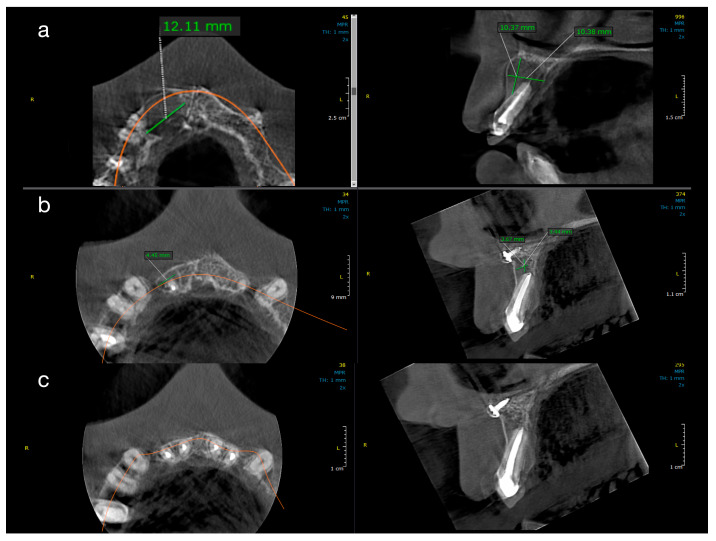
Sequential CBCT images of a case from Group 2 (decompression). Radiographic healing in a patient treated with root canal therapy and adjunctive decompression. (**a**) Baseline: critical-size lesion with dimensions of 12.11 mm (MD), 10.37 mm (BL), and 10.38 mm (V). (**b**) At 1 year: significant size reduction to 4.45 mm (MD), 3.67 mm (BL), and 3.84 mm (V). (**c**) At 2 years: residual lesion measuring ~3.67 mm in height and ~3.5 mm in width.

Figure 2 and Figure 3 illustrate representative cases from each group, highlighting the volumetric healing over time. In a Group 1 case (conventional treatment only), the initial lesion (~8–10 mm in diameter in each dimension) showed substantial shrinkage by the 1-year follow-up and almost complete radiological resolution by 2 years. By contrast, a Group 2 case (with decompression), which began with a larger cystic lesion (over 12 mm in its greatest dimension), demonstrated a dramatic reduction in lesion size after 12 months of decompression and continued healing at 24 months, leaving only a small residual radiolucency. These examples underscore the progressive radiographic healing observed in both treatment protocols.

All ten treated teeth remained asymptomatic throughout the follow-up, and sequential CBCT evaluations confirmed continuous lesion regression in every case. Table 1 and Table 2 detail the lesion size measurements (in mm) for each patient at baseline, 1 year, and 2 years in Group 1 and Group 2, respectively. Many lesions exhibited complete or near-complete radiological healing by the end of the observation period (recorded as 0.00 mm lesion diameter in at least one dimension for several cases in both groups). At baseline, Group 2 lesions were notably larger than those in Group 1. The mean initial lesion diameter in Group 2 was 13.7 mm (±3.34), compared to 7.3 mm (±1.74) in Group 1 (Table 3). This difference was statistically significant (*p* < 0.001), confirming that Group 2 started with more extensive periapical lesions. It is noteworthy that the baseline lesion size data in both groups showed no significant departures from normal distribution (see Table 4 for normality test results), which validated the use of parametric statistical comparisons.

**Figure 4:**
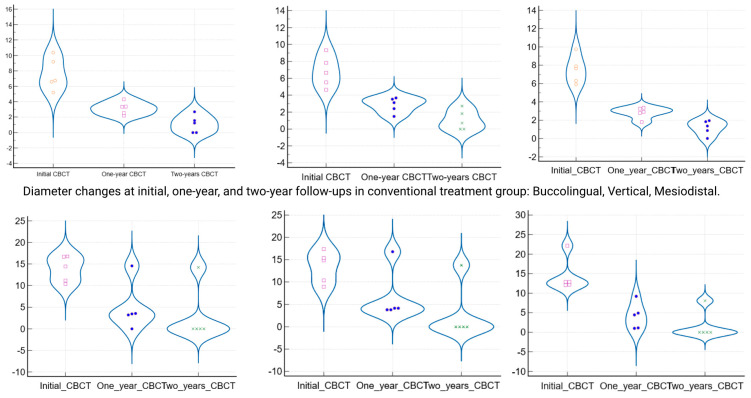
Violin plots of lesion size distribution in all three planes at baseline, 1 year, and 2 years (groups 1 and 2). Distribution of lesion dimensions (mesiodistal, buccolingual, and vertical) at baseline, 1-year, and 2-year follow-ups in both groups. Violin plots illustrate intergroup differences and the degree of variability over time. Group 2 showed greater initial lesion sizes and higher variance, especially at 1 year.

By the 12-month follow-up, the lesion dimensions had decreased markedly in both groups (Table 3). In Group 1 (standard endodontic therapy), the mean lesion diameter reduced from 7.3 mm at baseline to 2.93 mm (±0.75) at one year. In Group 2 (endodontic therapy + decompression), the mean lesion diameter decreased from 13.7 mm to 5.21 mm (±4.75) at one year. Although the lesions in Group 2 remained larger in absolute terms after 12 months, the difference between the two groups was no longer statistically significant (*p* = 0.087). Figure 4 presents violin plots of the lesion size distributions at baseline and one year, illustrating the substantial shift toward smaller lesion diameters in both cohorts. The spread of the violin plot for Group 2 at one year was broader, reflecting greater variability in healing outcomes among those larger initial lesions. By the 24-month follow-up, both groups showed further improvement. Group 1 lesions had a mean diameter of 1.12 mm (±0.98), and Group 2 lesions averaged 2.41 mm (±5.15). This slight residual difference was not statistically significant (*p* = 0.356). Radiographically, most lesions in both groups had resolved or nearly resolved by two years, with only very small radiolucencies, if any, remaining. It was observed that Group 2 continued to exhibit a higher variability in lesion size outcomes at 24 months (as evidenced by a larger standard deviation and wider confidence interval in Table 3). Importantly, within each group, the reduction in lesion size from baseline was highly significant. Both Group 1 and Group 2 demonstrated statistically significant lesion shrinkage at the 1-year and 2-year evaluations compared to baseline (intragroup comparisons, *p* < 0.001 for all). This confirms that both treatment approaches achieved effective and progressive lesion resolution over time in their respective groups.

**Figure 5:**
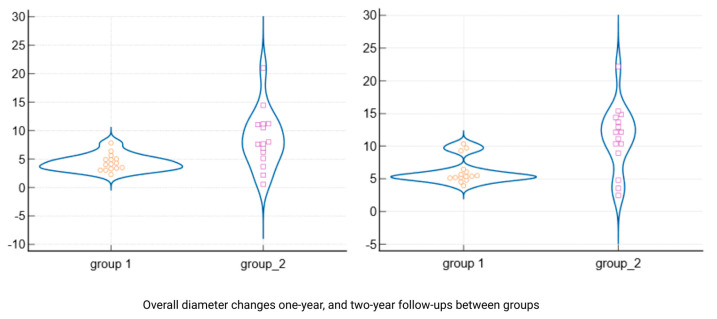
Violin plots show absolute reduction in lesion size at 1-year and 2-year follow-ups. Comparison of absolute lesion size reduction between groups after 1 year and 2 years of treatment. Group 2 (decompression) exhibited significantly greater mean reductions than Group 1 (*p* = 0.008 at 1 year, *p* = 0.002 at 2 years), despite larger initial lesion sizes.

When comparing the magnitude of lesion size reduction between the two protocols, the decompression-assisted approach yielded a greater overall change. Table 5 summarizes the absolute reduction in lesion diameter for each group at one year and two years. After one year of treatment, Group 2 lesions had shrunk by an average of 8.49 mm (±5.09), whereas Group 1 showed a mean reduction of 4.36 mm (±1.46). This difference in reduction was statistically significant (*p* = 0.008), indicating that the adjunctive decompression led to a faster and more pronounced shrinkage of the cystic lesions in the first year. A similar trend was noted at the end of the second year: Group 2 achieved a mean overall lesion size reduction of 11.29 mm (±5.02) from the baseline, compared to a 6.18 mm (±1.98) reduction in Group 1. The greater two-year reduction in Group 2 was also statistically significant (*p* = 0.002). The distributions of lesion reduction outcomes are visualized in Figure 5, which highlights the greater median shrinkage in the decompression group at both follow-up points. Notably, despite having larger initial lesions, the teeth treated with decompression not only “caught up” to the healing of the conventional cases but actually exhibited a more substantial absolute recovery in periapical bone fill over the two-year period.

In five of the Group 2 cases, periapical tissue samples were collected during the decompression procedure for histopathological analysis. The biopsy results confirmed the lesions to be radicular (periapical) cysts characterized by chronic inflammatory infiltrate and areas of fibrous tissue repair, aligning with the clinical and radiographic diagnosis of cystic lesions. In one decompression case, *Actinomyces* colonies were identified within the lesion—an unusual finding often associated with persistent extra-radicular infection. Importantly, even this case with actinomycosis responded favorably to the conservative treatment and healed without surgical intervention. Overall, the results after two years demonstrate that both nonsurgical strategies were effective in resolving extensive periapical lesions. However, the addition of decompression led to more rapid initial improvement and greater total reduction in lesion size, particularly evident within the first 12 months of follow-up. The two treatment groups had comparable outcomes by 24 months, with all affected teeth retained and showing radiographic signs of healing or complete bone regeneration. The data objectively support the benefit of the decompression-assisted approach in accelerating periapical lesion resolution while ultimately confirming that even large cyst-like lesions can be successfully managed with conservative endodontic methods.

In five cases from the decompression group, histopathological examination confirmed the presence of radicular cysts. All other lesions are referred to as periapical radiolucencies, reflecting the absence of histological confirmation.

## 4. Discussion

The benefit of performing endodontic treatment in the healing of periapical lesions lies in the removal of infected pulpal tissue and the significant reduction in bacteria within the root canal [19,20]. Various antimicrobial agents, such as sodium hypochlorite, are used in endodontics as irrigants to decontaminate and clean the endodontic space. These agents have a broad spectrum and nonspecific killing activity against bacteria, spores, and viruses [21], making endodontic therapy the primary option for treating periapical cysts. In a surgical procedure without endodontic therapy, the key factors contributing to the development of the lesion remain completely untreated [11]. Moreover, the use of calcium hydroxide as a root canal medication between appointments helps improve the disinfection of the endodontic system by neutralizing any remaining microorganisms, thus creating a conducive environment for periapical healing [22].

The study supports the conservative management of large cystic lesions using endodontic treatment, with or without decompression. Decompression enhanced initial healing in larger lesions by reducing intralesional pressure and facilitating drainage. This was clearly evident in Group 2, where lesions initially exceeding 10 mm showed more rapid volumetric reduction and radiographic resolution within the first year [14,15,23].

In Group 1, where only calcium hydroxide therapy was applied, healing progressed more gradually, yet resulted in comparable outcomes by the 24-month follow-up. This supports prior reports by Caliskan (2004) and Estrela et al. (2002), which showed that well-executed nonsurgical therapy can be effective even in cases involving large periapical pathology [7,9]. The CBCT-based dimensional analysis confirmed lesion shrinkage in all three spatial planes, reinforcing the utility of CBCT not only for initial diagnosis but also for monitoring treatment response [23].

Recent studies further emphasize that nonsurgical strategies are not only effective but also cost-efficient and preserve periapical structures, reducing the need for apical surgery [24,25]. Similarly, Kumar et al. (2013) reported high success rates in managing periapical lesions conservatively, especially when calcium hydroxide was used for an extended period [26]. Additionally, well-monitored conservative therapy can achieve bone regeneration and maintain periapical integrity without surgical intervention [23].

Histopathological examination, performed on biopsies from five cases, confirmed the presence of radicular cysts with chronic inflammatory infiltrate and areas of fibrous repair. Notably, one case revealed actinomycosis, a rare and persistent extra-radicular infection. Although endodontic therapy alone is often reported to be insufficient for healing such infections [27,28], in this case, complete resolution was achieved using a conservative protocol with decompression. This outcome highlights the potential of decompression to aid in managing otherwise treatment-resistant periapical pathologies. These findings emphasize the need for differential diagnosis in large lesions, as clinical and radiological features alone may not sufficiently distinguish between cysts and granulomas [3,29].

Importantly, none of the cases required apical surgery or extraction during the follow-up period, highlighting the potential of conservative protocols to preserve teeth that might traditionally have been considered for surgical intervention [30]. Our data add to the growing evidence base advocating for tailored, minimally invasive approaches in endodontic therapy, especially when CBCT and histopathology are employed judiciously for comprehensive assessment.

A nonsurgical approach should always be the preferred treatment for endodontic-origin cystic lesions, including true cysts [11,31,32]. Decompression and aspiration–irrigation techniques are recommended only for draining cystic fluid when the moisture inside the root canals cannot be controlled [33]. These methods help the endodontist dry the root canal and enable the three-dimensional filling of the endodontic system [34].

A further limitation of this study is the inability to differentiate between true cysts (completely enclosed by epithelium) and bay cysts (communicating with the root canal) at baseline. Such classification requires histopathological confirmation, which was available only in the decompression group. As CBCT cannot reliably distinguish between these subtypes, all lesions were categorized radiographically as periapical radiolucencies. However, according to Ricucci’s findings [4], there is no need to distinguish between the two cyst types. Moreover, both types were shown to be associated with intra-radicular infection and to respond favorably to conservative endodontic treatment [4,11].

## 5. Conclusions

This study demonstrates that both conventional nonsurgical endodontic therapy and the same protocol supplemented by decompression are effective in managing large periapical cyst-like lesions over a two-year follow-up. While both groups achieved comparable healing at 24 months, decompression accelerated early lesion size reduction, particularly in extensive cases.

These findings support decompression as a minimally invasive adjunct for improving short-term outcomes in critical-size lesions, potentially avoiding surgical intervention and enhancing treatment predictability. CBCT proved valuable for longitudinal assessment, and histopathology, when available, refined diagnostic accuracy and guided decision-making.

## Figures and Tables

**Table 1:** CBCT measurements of lesion dimensions at baseline, 1 year, and 2 years (Group 1: conventional treatment).

Case	Measurement	Initial CBCT	1-Year CBCT	2-Year CBCT
Case 1	Mesiodistal	9.75	3.36	0.00
	Buccolingual	10.36	2.51	0.00
	Height (Vertical)	9.31	3.68	0.00
Case 2	Mesiodistal	6.29	3.26	0.87
	Buccolingual	6.74	3.36	1.54
	Height (Vertical)	4.65	2.40	0.71
Case 3	Mesiodistal	5.90	1.80	1.37
	Buccolingual	5.20	2.16	0.00
	Height (Vertical)	5.50	1.49	0.00
Case 4	Mesiodistal	7.89	2.91	1.85
	Buccolingual	6.58	3.32	1.24
	Height (Vertical)	7.82	3.53	2.72
Case 5	Mesiodistal	7.65	2.78	1.96
	Buccolingual	9.17	4.30	2.69
	Height (Vertical)	6.65	3.13	1.82

**Table 2:** CBCT measurements of lesion dimensions at baseline, 1 year, and 2 years (Group 2: decompression group).

Case	Measurement	Initial CBCT	1-Year CBCT	2-Year CBCT
Case 1	Mesiodistal	12.18	1.02	0.00
	Buccolingual	14.42	0.00	0.00
	Height (Vertical)	8.93	3.80	0.00
Case 2	Mesiodistal	22.15	1.15	0.00
	Buccolingual	11.16	3.55	0.00
	Height (Vertical)	15.36	4.15	0.00
Case 3	Mesiodistal	12.11	4.45	0.00
	Buccolingual	10.38	3.44	0.00
	Height (Vertical)	10.37	4.15	0.00
Case 4	Mesiodistal	12.88	9.19	8.08
	Buccolingual	16.72	14.56	14.23
	Height (Vertical)	17.37	16.79	13.80
Case 5	Mesiodistal	12.90	4.89	0.00
	Buccolingual	13.69	3.18	0.00
	Height (Vertical)	14.85	3.79	0.00

**Table 3:** Summary of mean lesion sizes at baseline, 1 year, and 2 years in both groups (all dimensions combined).

	Initial CBCT Group 1	One-Year CBCT Group 1	Two-Years CBCT Group 1	Initial CBCT Group 2	One-Year CBCT Group 2	Two-Years CBCT Group 2
Mean	7.3	2.93	1.12	13.7	5.21	2.41
Std. Deviation	1.74	0.75	0.98	3.34	4.75	5.15
Minimum	4.65	1.49	0	8.93	0	0
Maximum	10.36	4.3	2.72	22.15	16.79	14.23
95% CI	6.33–8.26	2.52–3.35	0.57–1.66	11.85–15.55	2.58–7.84	−0.44–5.26
Mean ± Std.	7.3 ± 1.74	2.93 ± 0.75	1.12 ± 0.98	13.7 ± 3.34	5.21 ± 4.75	2.41 ± 5.15

**Table 4:** Normality tests for baseline lesion sizes in groups 1 and 2.

	Initial CBCT Group 1	Initial CBCT Group 2	*p* Value	*p* Value
Mean	7.3	13.7	0.789 ^a^	0. 941 ^a^
Std. Deviation	1.74	3.34	0.627 ^b^	0.353 ^b^
Minimum	4.65	8.93	0.590 ^c^	0.502 ^c^
Maximum	10.36	22.15		
Mean ± Std.	7.3 ± 1.74	13.7 ± 3.34		

^a^ Kolmogorov–Smirnov, ^b^ Shapiro–Wilk, ^c^ Anderson–Darling.

**Table 5:** Absolute reduction in lesion diameter at 1-year and 2-year follow-ups in both groups.

	ONE YEAR		TWO YEARS	
	GROUP 1	GROUP 2	GROUP 1	GROUP 2
Mean	4.36	8.49	6.18	11.29
Std. Deviation	1.46	5.09	1.98	5.02
Minimum	2.25	0.58	3.94	2.49
Maximum	7.85	21	10.36	22.15
95% Confidence interval for mean	3.56–5.17	5.67–11.31	5.08–7.27	8.51–14.07
Mean ± Std.	4.36 ± 1.46	8.49 ± 5.09	6.18 ± 1.98	11.29 ± 5.02

## Data Availability

The data supporting the findings of this study are available upon request.
